# Coverage of the WHO’s four essential elements of newborn care and their association with neonatal survival in southern Nepal

**DOI:** 10.1186/s12884-020-03239-6

**Published:** 2020-09-16

**Authors:** Emily Bryce, Luke C. Mullany, Subarna K. Khatry, James M. Tielsch, Steven C. LeClerq, Joanne Katz

**Affiliations:** 1 Department of International Health, Johns Hopkins Bloomberg School of Public Health, 615 N. Wolfe Street, Baltimore, MD 21205 USA; 2 Nepal Nutrition Intervention Project - Sarlahi (NNIPS), Nepal Eye Hospital Complex, PO Box 335, Tripureshwor, Kathmandu, Nepal; 3 Department of Global Health, Milken Institute School of Public Health, George Washington University, 950 New Hampshire Avenue, Washington, DC, USA

**Keywords:** Neonatal mortality, Immediate drying, Skin to skin contact, Cord clamping, Early initiation of breastfeeding

## Abstract

**Background:**

Despite recent improvements in child survival, neonatal mortality continues to decline at a slower rate and now represents 47% of under-five deaths globally. The World Health Organization developed core indicators to better monitor the quality of maternal and newborn health services. One such indicator for newborn health is “the proportion of newborns who received all four elements of essential care”. The four elements are immediate and thorough drying, skin to skin contact, delayed cord clamping, and early initiation of breastfeeding. Although there is existing evidence demonstrating an association with decreased neonatal mortality for each element individually, the cumulative impact has not yet been examined.

**Methods:**

This analysis uses data from a randomized trial to examine the impact of sunflower versus mustard seed oil massage on neonatal mortality and morbidity in the Sarlahi district in Southern Nepal from 2010 to 2017. The proportion of newborn infants receiving an intervention was the exposure and neonatal mortality was the outcome in this analysis. Neonatal mortality was defined as a death between three hours and less than 28 days of age. Associations between neonatal mortality and the essential elements were estimated by Cox proportion hazards models. The hazard ratios and corresponding 95% confidence intervals were reported.

**Results:**

28,121 mother-infant pairs and 753 neonatal deaths were included. The percent receiving the individual elements ranged from 19.5% (skin to skin contact) to 68.2% (delayed cord clamping). The majority of infants received one or two of the elements of essential care, with less than 1% receiving all four. Skin to skin contact and early initiation of breastfeeding were associated with lower risk of neonatal mortality (aHR = 0.64 [0.51, 0.81] and aHR = 0.72 [0.60, 0.87], respectively). The risk of mortality declined as the number of elements received increased; receipt of one element compared to zero was associated with a nearly 50% reduction in risk of mortality and receipt of all four elements resulted in a 72% decrease in risk of mortality.

**Conclusions:**

The receipt of one or more of the four essential elements of newborn care was associated with improved neonatal survival. The more elements of care received, the more survival improved.

## Background

Between 1990 and 2017 the global under-five mortality rate declined by 58% and the neonatal mortality rate by 51% [1]. Despite this progress, neonatal mortality continues to decline at a slower rate than under-five mortality and both measures remain well above the Sustainable Development Goal (SDG) targets for 2030 [1, 2]. Nepal was one of the few countries that achieved the antecedent Millennium Development Goal’s (MDG) fourth target, with the under 5 mortality rate declining from 162 to 38 deaths per 1000 live births. During the same time period, neonatal mortality in Nepal declined from 39 to 23 deaths per 1000 live births [3, 4].

In an effort to continue the progress made in reducing maternal, child and neonatal mortality, the global community has recognized that in going forward with the SDGs there needs to be a focus on quality of care measurement [5]. For this purpose, the World Health Organization (WHO) developed and released a list of global indicators for maternal and newborn care in 2014, one of which is the “proportion of newborns who received all four elements of essential care”, which are as follows: immediate and thorough drying, immediate skin-to-skin contact, delayed cord clamping and initiation of breast feeding within the first hour [6].

Immediate and thorough drying (ITD) of the infant serves two purposes in reducing neonatal mortality: aiding in stimulation/resuscitation and thermal regulation of infants [7]. Roughly 10 million babies annually will require simple stimulation, which includes ITD, in order to begin breathing [8]. Neonatal hypothermia has been shown to be associated with higher risk of mortality and morbidity, particularly in preterm and low birthweight infants [9–11]. The primary source of heat loss for a newborn is the evaporation of amniotic fluid from the baby’s skin. Often, a newborn’s temperature drops 3–4 °C within minutes after being born [12]. This almost immediate change in temperature highlights the importance of ITD to reduce the presence of amniotic fluid on the baby’s body.

Skin to skin contact (SSC) is important for establishing breastfeeding practices, thermal regulation of a newborn, and transfer of normal skin flora from mother to infant. Infants who experienced SSC were more likely to be breastfed one to four months postpartum, to be exclusively breastfed at six weeks to six months, and breastfeed more effectively [13, 14]. The second most common source of heat loss for a newborn is via conduction, or being placed on a cold surface [12]. Immediate SSC ensures that the baby is placed on a warm surface, their mother’s chest. SSC is recommended for hypothermia prevention and has been shown to be at least as effective in temperature maintenance in newborns as conventional incubators [12, 15].

The third element in the WHO’s package of essential elements for newborn care is delayed cord clamping (DCC), which is defined as the severing of the umbilical cord at least one minute after delivery or when the umbilical cord is no longer pulsating [16]. DCC allows the newborn to continue receiving fetal blood from the placenta after delivery, which can result in 60% more red blood cells and a 30% increase in blood volume [17]. Other suggested benefits of DCC include increased hemoglobin concentrations, reduced rates of anemia, improved cardiopulmonary adaptation, and better iron status in infants six months postpartum [17, 18].

Early initiation of breastfeeding (EIB) is defined as initiation of breastfeeding within the first hour after delivery. There is substantial evidence demonstrating that breastfeeding, specifically exclusive breastfeeding for six months, is a cheap and effective way to reduce all-cause and infection-related infant mortality [19]. Recently, there has been a shift in focus to the timing of breastfeeding initiation. Early initiation has been shown to result in an increased duration of and exclusivity of breastfeeding [20, 21]. Furthermore, beginning to breastfeed within the first hour also ensures that the baby is exposed to colostrum, which is associated with improved development of the intestinal mucosal and immunological protections [22].

The logic is that neonates who receive all four essential elements are more likely to survive than those who do not, given the pre-existing evidence base for the individual elements. As the indicator was recently developed, there has been limited research conducted looking at the four elements together. This paper will examine the current coverage of each individual element, distribution of number of elements received, associated characteristics with receipt of each element, and the association(s) between the element(s) and neonatal mortality in Sarlahi district of Nepal.

## Methods

### Parent trial

The data used for this analysis were originally collected as part of a cluster-randomized community based trial (ClinicalTrials.gov, NCT01177111) on the impact of sunflower seed oil versus standard of care mustard seed oil massage on neonatal mortality and morbidity. This study, conducted in Sarlahi district, Nepal, consented and enrolled incident pregnancies and followed the women through delivery and 28 days postpartum [23]. Data on the four elements were collected through post-partum interviews and neonatal mortality data through verbal autopsy interviews, 75% of which were with the mothers. In those instances, the neonatal verbal autopsy interview was completed with a family member These interviews were conducted as soon as possible and appropriate (death) after the event. About 80% were visited within 72-h of birth and 80% within a month of death.. Maternal characteristics were collected at enrollment in the parent study.

### Analysis overview

The element “immediate and thorough drying” was defined as whether the baby was completely wiped with a cloth prior to placental delivery. An infant was considered to have received the “immediate skin to skin contact” element if they were placed on the mother’s chest and/or in her arms before delivery of the placenta. In the parent trial, exact time to cord clamping was not collected, therefore for this analysis “delayed cord clamping” was defined as clamping the umbilical cord after the placenta was delivered. “Early initiation of breastfeeding” was defined as the infant initiating breastfeeding within the first hour postpartum. Associations between maternal-infant characteristics and receipt of the four elements were examined using log binomial regression.

The main outcome of interest was neonatal mortality defined as a death between 3 h and 28 days of age. To account for reverse causality, infants who died within 3 h of birth were removed from the analysis, as it is possible that their status immediately postpartum may have impacted the likelihood of receiving the four essential elements. A total of 28,121 mother-infant pairs where the infant survived at least three hours postpartum and 753 neonatal deaths were included in the analysis. The choice of 3 h as the cut off was arbitrary, but a sensitivity analysis was conducted using 2 and 4 h cut offs and found that this did not significantly change the estimated hazard ratios for any of the four elements (Supplemental Table 1). For the same reason, multiple births and mother-infant dyads where the mother died during childbirth were also removed from the analysis. Therefore, singleton, live-born infants that survived beyond three hours postpartum and their mothers who survived childbirth were included in the analysis.

To examine the relationship between an individual element and neonatal mortality, all observations for the element were included in the analysis, irrespective of values of the other elements (received, not received, or missing). For analyses where all four elements are included in the model, only those infant-mother pairs with no missing data on the four elements are included. The same is true for the analyses examining number of elements received (0–4) and mortality, as to not assume that a “don’t know” response means that the infant did not receive the element.

As a sensitivity analysis, mortality was examined for infants who survived more than two days. The restriction to at least two-day survival is of interest because all four interventions may not have an immediate effect on mortality, rather the impact may be seen in longer-term survival rates. This also allowed for the removal of a more extreme reverse causality potentially present in this analysis.

Coverage of each element was calculated, as was the distribution of infants receiving none, one, two, three and all four of the elements. Coverage was defined as all of those in need of an intervention (in the analysis, live born singleton infants) who received an intervention (the essential elements) [24].

Mortality was examined using live births that survived beyond three hours postpartum as the denominator and deaths between three hours and 28 days as the numerator. Mortality rates for infants who received each element were reported, as well as hazard ratios estimated using a Cox proportional hazard model (with the Efron method for ties) to determine the relationship between mortality and the four elements after controlling for potential confounders. The covariates included sex of infant, preterm birth (< 37 weeks gestation), mother’s literacy, a socioeconomic composite score divided into quartiles, parity (first birth vs. not) and whether the birth took place in a facility.

The DDC and EIB elements violated the proportional hazards assumption for the Cox model, assessed by Schoenfeld residual plots and proportionality tests. To correct for this, the analyses for these two elements were stratified by time, less than three days postpartum and greater than three days postpartum. The model with all four elements included was also stratified to maintain the proportional hazards assumption.

All analyses were conducted using Stata version 14.2 (Stat Corp, College Station, Texas). The Institutional Review Board of the Johns Hopkins Bloomberg School of Public Health (Baltimore, Maryland) and the Ethical Review Committee of the Institute of Medicine, Tribhuvan University (Kathmandu, Nepal) approved the parent study.

## Results

### Study population

A total of 28,121 mother-infant pairs where the singleton infant survived at least three hours postpartum were included in the analysis, resulting in a total analysis time of 757,605 days. There were 753 neonatal deaths (death after 3 h and by 28 days postpartum), resulting in an overall neonatal mortality rate for infants surviving beyond three hours postpartum of 26.8 neonatal deaths per 1000 live births. Were 814 infants that were lost to follow up during the 28-day period, equaling 11,383 days lost to follow-up.

Table 1 summarizes the characteristics of the mother-infant pairs in the analysis set. Comparing facility and non-facility births, a higher proportion of facility-born infants received ITD, SSC and EIB. However, 98.2% of non-facility births received DCC, compared to only 22.1% of facility births. A higher proportion of preterm infants died than non-preterm infants. A separate analysis (Supplemental Table 2) examined relationships between characteristics and element receipt and found facility birth, mother’s literacy and parity to have the strongest associations.

**Table 1:** Characteristics of mother-infant pairs

	All mother-infant pairs n(%) *N* = 28,121	Immediate and thorough drying n(%) *N* = 10,914	Skin to skin contact n(%) *N* = 5324	Delayed cord clamping n(%) *N* = 18,252	Early initiation of breastfeeding n(%) *N* = 8459	Mortality n (%) *N* = 753
Sex of infant						
Male	14,528 (51.7%)	5723 (41.3%)	2872 (20.4%)	9225 (66.8%)	4583 (31.6%)	401 (2.8%)
Female	13,593 (48.3%)	5191 (39.8%)	2452 (18.54%)	9027 (69.6%)	3876 (28.5%)	352 (2.6%)
Preterm birth						
> 37 weeks	22,698 (81.0%)	8934 (41.1%)	4483 (20.3%)	14,524 (67.2%)	6976 (30.8%)	442 (1.9%)
< 37 weeks	5324 (19.0%)	1944 (38.3%)	821 (15.9%)	3653 (72.2%)	1455 (27.4%)	306 (5.8%)
Mother’s literacy						
Literate	8814 (31.3%)	3872 (47.2%)	2378 (28.3%)	4417 (54.3%)	3097 (35.2%)	198 (2.3%)
Illiterate	19,300 (68.7%)	7038 (37.7%)	2943 (15.6%)	13,832 (74.2%)	5359 (27.8%)	555 (2.9%)
SES quartile						
First	9609 (34.2%)	3445 (36.9%)	1332 (14.1%)	7132 (76.4%)	2613 (27.2%)	286 (3.0%)
Second	6056 (21.5%)	2352 (40.4%)	1067 (18.1%)	3965 (68.4%)	1796 (29.7%)	174 (2.9%)
Third	5773 (20.5%)	2265 (41.2%)	1216 (21.7%)	3579 (65.6%)	1767 (30.7%)	135 (2.3%)
Fourth	6683 (23.8%)	2852 (45.87%)	1709 (26.9%)	3576 (57.8%)	2283 (34.2%)	158 (2.4%)
Parity						
Nulliparous	7886 (28.0%)	3383 (46.1%)	1944 (25.9%)	4096 (56.3%)	2256 (28.6%)	279 (3.5%)
Multiparous	20,234 (72.0%)	7531 (38.6%)	3379 (17.1%)	14,156 (72.6%)	6203 (30.7%)	474 (2.3%)
Place of delivery						
Hospital/clinic	11,875 (42.2%)	6376 (59.6%)	5021 (45.3%)	2341 (22.1%)	4876 (41.1%)	355 (3.0%)
At home/other	16,245 (57.8%)	4537 (28.1%)	303 (1.9%)	15,911 (98.2%)	3583 (22.1%)	398 (2.5%)

### Coverage

**Fig. 1:**
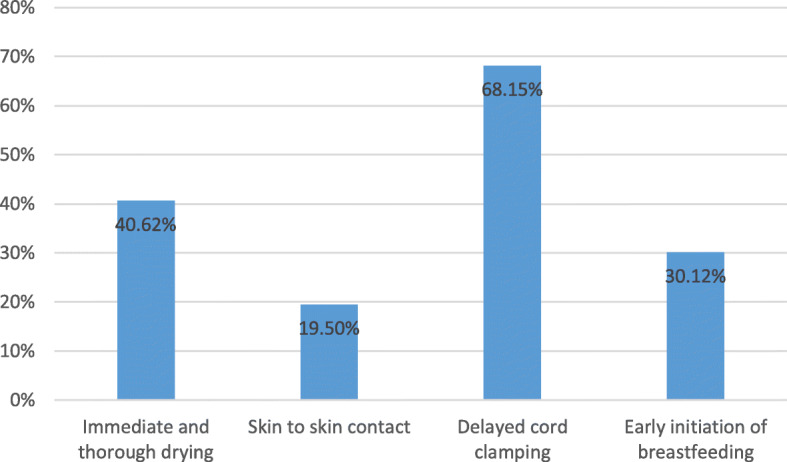
Coverage of each WHO essential element (%)

The element coverage is displayed in Fig. 1. Delayed cord clamping had the highest coverage (68.2%) and skin to skin contact has the lowest (19.5%). The majority of infants received one or two elements, 48.1 and 37.2%, respectively (Table 2). 11.0% of infants received three of the elements and 0.9% received all four. Finally, 2.8% of infants received none of the essential elements.

**Table 2:** Distribution of four elements in population

Number of WHO essential elements of newborn care received	Coverage (%)	Total (n)
0	2.8%	736
1	48.1%	12,638
2	37.2%	9783
3	11.0%	2911
4	0.9%	227

### Essential element(s) association(s) with neonatal mortality

Bivariate Cox proportional hazards analyses examined the unadjusted associations of the elements with neonatal mortality and showed a protective effect for two of the four elements (Table 3). EIB was associated with the largest reduction in risk of neonatal mortality (HR = 0.68 [0.56, 0.82]). SSC was associated with 22% reduction in risk of mortality.

**Table 3:** Mortality risk by essential element, unadjusted and adjusted^a^

Indicator	n	Died, n	Mortality Rate	HR	95% CI	aHR	95% CI
Immediate and thorough drying				1.11	(0.95, 1.29)	1.09	(0.93, 1.27)
Received	10,914	295	27.0 per 1000 live births				
Did not receive	15,954	390	24.4 per 1000 live births				
Skin to skin contact				0.78*	(0.64, 0.95)	0.64**	(0.51, 0.81)
Received	5324	113	21.2 per 1000 live births				
Did not receive	21,980	596	27.1 per 1000 live births				
Delayed cord clamping				1.05	(0.90, 1.23)	0.87	(0.66, 1.14)
Received	18,252	425	23.3 per 1000 live births				
Did not receive	8530	247	29.0 per 1000 live births				
Early Initiation of Breastfeeding				0.68**	(0.56, 0.82)	0.72**	(0.60, 0.87)
Received	8459	139	16.4 per 1000 live births				
Did not receive	19,624	614	31.3 per 1000 live births				

*p < 0.05

**p < 0.01

^a^covariates (PTB, sex of infant, mother’s literacy, SES, parity and place of delivery)

Multivariable survival analyses were conducted for all infants who survived past three hours postpartum for the four elements separately (Table 3, all variables in Supplemental Table 3). Adjusting for potential confounders, three of the four elements were associated with a reduction in mortality risk. The hazard ratio associated with EIB was slightly attenuated after adjustment, to a 28% reduction in risk of neonatal mortality. The magnitude of the HR for SSC increased (aHR = 0.64 [0.51, 0.81]). A small positive association between ITD and neonatal mortality remained after adjustment, though it is statistically insignificant. The separate analysis for infants who survived past 48-h (Supplemental Table 5) attenuated the associations of two of the elements with risk of mortality; SSC remained the only significant element with an aHR = 0.66 [0.49, 0.90]. The DCC-mortality association qualitatively changed, now positive in the post-48-h survival group along with ITD.

The four elements were found to be correlated with one another (Supplemental Table 5, correlation range [− 0.4953, 0.1493]). Therefore, a model was fit to include all elements to assess the impact of each element controlling for the others in the three-hour and the 48-h survival groups (Supplemental Table 6). However, for ITD and DCC the adjustment for the other elements had little to no effect on the point estimates compared to the single-element models in both survival-time groups. The point estimates for SSC and EIB were attenuated slightly, a relative ~ 10% decrease in estimated hazard ratios.

Risk of neonatal morality associated with each element was stratified by preterm birth status, given its significance in the single element models and its known association with mortality (Supplemental Table 7). All four elements demonstrated a protective effect against neonatal mortality in the preterm infants, however these associations were not significant, likely due to sample size limitations.

### Number of elements received and associated mortality

**Fig. 2:**
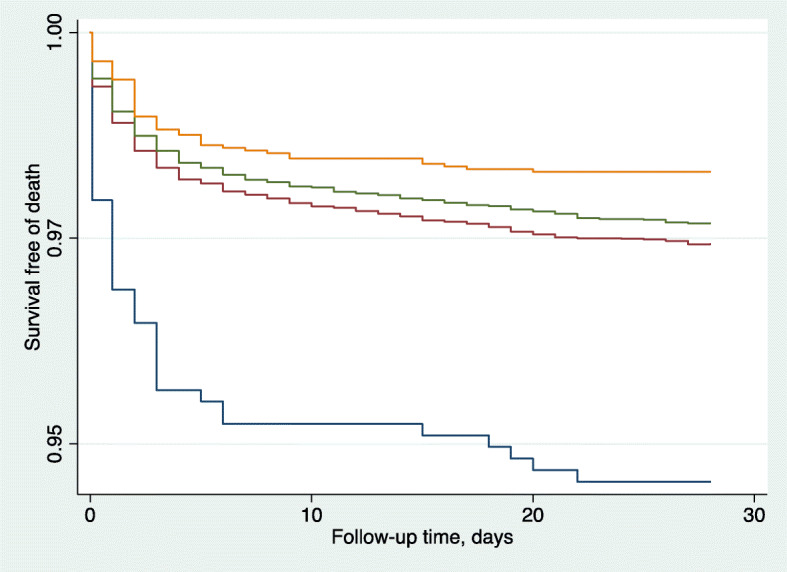
WHO essential element Kaplan-Meier survival estimates. Received 0 (blue); Received 1 (red); Received 2 (green); Received 3 or 4 (orange)

The association between the number of essential elements received and neonatal mortality (as defined as a death between three hours and less than 28 days) was also examined through bivariate and multivariable Cox proportional hazards models (Table 4, Fig. 2). Both analyses demonstrate that the associated risk of mortality decreased as the number of essential elements received increased. Compared to infants who received no essential elements, receiving one element was associated with a nearly 50% decrease in mortality. Receipt of two and three elements had an even larger reduction in mortality risk (aHR = 0.46 and aHR = 0.34, respectively). Infants who received all four elements had the largest reduction in risk of mortality (72%) compared to infants who received none. However, very few infants received all four elements in this population.

**Table 4:** Unadjusted and adjusted^a^ mortality by distribution of elements in infants surviving more than three hours

	N	Mortality	HR	95% CI	aHR	95% CI
Received zero of the four elements	736	40	Ref	Ref	Ref	Ref
One element	12,638	324	0.46**	(0.33, 0.64)	0.51**	(0.36, 0.71)
Two elements	9783	226	0.42**	(0.30, 0.58)	0.46**	(0.33, 0.65)
Three elements	2911	50	0.31**	(0.20, 0.47)	0.34**	(0.23, 0.52)
Four elements	227	3	0.24*	(0.07, 0.76)	0.28*	(0.09, 0.89)

*p < 0.05

**p < 0.01

^a^covariates (PTB, sex of infant, mother’s literacy, SES, parity and place of delivery)

## Discussion

This analysis demonstrated that the greater the number of WHO’s elements of essential newborn care that an infant receives, the lower their associated risk of mortality. The analysis also showed that despite these elements being recommended by the WHO, their coverage is quite variable in this population in rural Southern Nepal.

In the adjusted models, skin to skin contact, delayed cord clamping, and early initiation of breastfeeding were all associated with lower risks of mortality, whereas immediate and thorough drying was associated with a slight increased risk of mortality. As ITD is often included in a package of practices for neonatal resuscitation, there are few papers examining this single element’s impact on neonatal mortality. One study that examined the 2006 WHO recommendations for newborn care using data from the 2011 Bangladesh DHS also found an increased, but statistically insignificant, risk of neonatal mortality associated with drying within five minutes of delivery (OR = 1.64, 0.75, 3.58) [25]. A possible explanation for the increased associated risk of death for this element is that infants who were having difficulty breathing were more likely to receive ITD as part of the neonatal resuscitation procedure. Infants with issues breathing initially have been shown to be at a higher risk for mortality, confounding the impact for this element [26]. In the stratified analysis, ITD was associated with an insignificant reduction in mortality for preterm infants. As demonstrated by Mullany et al., preterm infants are at the highest risk of hypothermia-related mortality which is the other pathway through which ITD is hypothesized to offer a protective effect [26].

Skin to skin contact and early initiation of breastfeeding were consistently associated with a reduction in neonatal mortality in this analysis. There is a substantial amount of evidence supporting EIB as a simple, effective tool to reduce neonatal mortality, consistent with the findings presented here [22]. Skin to skin contact as part of kangaroo mother care has been shown to reduce mortality in low birthweight infants as well as improve breastfeeding practices [13, 27, 28]. In the model that included all four elements, both SSC and EIB demonstrated a significant reduction in risk of neonatal mortality (defined as death between three hours and 28 days postpartum) after controlling for the other elements and covariates, indicating that despite the relationship between the two elements, they both offer protective effects independent of the other.

The association between risk of mortality and delayed cord clamping was inconsistent across the models. This is congruent with other findings in both term and preterm infants, none of which found significant impacts on mortality associated with delayed cord clamping. Those analyses reported that in preterm infants, delayed cord clamping was associated with a significant decrease in the need for transfusions, intraventricular hemorrhage and necrotizing enterocolitis [17, 29]. These outcomes were not assessed in this study, but may be considered in recommendations in the future.

The study’s strengths are the size, completeness, short recall period and regular follow-up schedule of the parent trial. Additionally, the amount of information collected allowed for adjusting for multiple potentially confounding variables. Furthermore, surveys were conducted as soon as appropriate after births and deaths of infants to reduce potential recall bias.

The data for this analysis was collected using primarily maternal recall (few instances of family member recall for sections of the newborn verbal autopsy questionnaire), which is a potential limitation because maternal recall of events around labor and delivery has been shown to have varied validity. Previous validation studies that assessed maternal recall of three elements (DCC not examined) did not have high accuracy (AUC > 0.70); the AUCs ranged from 0.50–0.63, indicating poor to moderate validity [30–32]. These studies were conducted in facilities where the validity of these recall measures may have been worse than for home births where the mother may have had more ability to see and know what was happening to the infant right after birth. In this study, the elements DCC and ITD had the highest numbers of “don’t know” responses (Supplemental Table 8), both of which a woman is likely to know if it occurred if she is told by a health care provider or family member present at delivery. However, there was no assessment of the validity of the maternal recall for home versus facility births in the parent study.

According to two reports, information on these elements collected in household and facility surveys to measure coverage is sparse, if collected at all [6, 33]. The fact that the parent trial collected information on these elements allowed us to demonstrate that the four elements together have the potential to make a sizable impact on neonatal mortality. Even though the magnitude of the reduction was not necessarily significant for all elements individually, there was a demonstrated dose response relationship between the number of the four elements received and reduced risk of mortality. Therefore, routine data on the coverage of the four elements, individually and the number received per infant, should be collected to inform programmatic efforts and health systems. This routine reporting of the four elements can act as a status update for health system functioning and goals for coverage can be set in order to reach the SDG’s neonatal mortality target of 12 deaths per 1000 live births [2].

As the majority of the 75 countries did not reach the MDG goals for child survival, it is apparent that further action is needed to achieve the SDGs. Determining how interventions interact, rather than exclusively studying them in isolation, can inform design and implementation of health programs. The results of this study demonstrate that these four interventions are associated with improved outcomes for newborns in a dose response way. Furthermore, these findings support designing future programs for newborn care with a multidimensional approach in order to improve newborn and child survival.

## Supplementary information

**Additional file 1.** Supplementary materials (Supplementary Tables 1–8, Supplementary Figs. 1, 2a-2d).

## Data Availability

The datasets used for the analysis are available from the corresponding author upon reasonable request.

## Abbreviations

DCC: Delayed cord clamping
EIB: Early initiation of breastfeeding
HR: Hazard Ratio
ITD: Immediate and thorough drying
MDG: Millennium Development Goal
SDG: Sustainable Development Goal
SSC: Skin to skin contact
WHO: World Health Organization
