# Supplementary material for: Coverage of the WHO’s four essential elements of newborn care and their association with neonatal survival in southern Nepal
Source: BMC Pregnancy Childbirth. 2020 Sep 16;20:540. doi: 10.1186/s12884-020-03239-6 (PMC7493414; doi:10.1186/s12884-020-03239-6)
Supplement: Supplementary file 1 — Additional file 1. Supplementary materials (Supplementary Tables 1–8, Supplementary Figs. 1, 2a-2d). [file 12884_2020_3239_MOESM1_ESM.docx]

**SUPPLEMENTARY MATERIALS**

Table 1. Adjusted Hazard Ratios by Different Mortality Time Cut Off

Table 2. Association between maternal-infant characteristics and receipt of elements of essential care…2

Figure 1. Number of WHO elements received…………………………………………………………….3

Table 3. Adjusted mortality by essential element for all infants, survival > 3 hours………………….…..3

Table 4. Adjusted mortality by essential element for all infants, survival > 48 hours….............................4

Table 5. Correlation matrix for four elements………………………………………………………….….5

Table 6. All elements adjusted for covariates and other elements, by survival time………………..…….5

Table 7. Adjusted mortality for all infants > 3 hours postpartum, by preterm birth……………………....5

Figures 2a-2d. Kaplan-Meier graphs for each of the four elements……………………………………….6

Table 8. Characteristics of mother-infant dyads with “missing” element information……………………8

**Supplemental Table 1. Adjusted^b^ Hazard Ratios by Different Mortality Time Cut Off**

|  | 2 hour cut off | 3 hour cut off | 4 hour cut off |
| --- | --- | --- | --- |
| ITD | 1.12 (0.96, 1.32) | 1.09 (0.93, 1.27) | 1.09 (0.93, 1.29) |
| DCC | 0.85 (0.65, 1.11) | 0.87 (0.66, 1.14) | 0.89 (0.68, 1.16) |
| SSC | 0.64 (0.50, 0.80) | 0.64 (0.51, 0.81) | 0.64 (0.51, 0.81) |
| EIB | 0.73 (0.61, 0.89) | 0.72 (0.60, 0.87) | 0.73 (0.61, 0.88) |

^b^covariates (PTB, sex of infant, mother’s literacy, SES, parity and place of delivery)

**Supplementary Table 2. Association between maternal-infant characteristics and receipt of elements of essential care**

| Indicator | RR | 95% CI | RR | 95% CI | RR | 95% CI | RR | | 95% CI |
| --- | --- | --- | --- | --- | --- | --- | --- | --- | --- |
|  | **Immediate and thorough drying** | | **Skin to skin contact** | | **Delayed cord clamping** | | **Early initiation of breastfeeding** | | |
| Sex of infant  (ref: male) | 0.98 | (0.96, 1.01) | 0.96 | (0.92, 1.00) | 1.01 | (0.98, 1.04) | 0.92** | (0.89, 0.96) | |
| Preterm Birth  (ref: >37 weeks) | 0.97 | (0.94, 1.01) | 0.90** | (0.85, 0.95) | 1.01 | (0.98, 1.05) | 0.93** | (0.89, 0.98) | |
| Mother’s literacy  (ref: illiterate) | 1.04** | (1.01, 1.08) | 1.08** | (1.03, 1.13) | 0.97 | (0.93, 1.00) | 1.10** | (1.06, 1.14) | |
| SES score  (ref: first quartile) |  |  |  |  |  |  |  |  | |
| Second quartile | 1.01 | (0.97, 1.04) | 0.98 | (0.92, 1.04) | 0.99 | (0.97, 1.04) | 1.01 | (0.96, 1.06) | |
| Third quartile | 0.99 | (0.95, 1.03) | 1.08** | (1.02, 1.15) | 0.99 | (0.95, 1.03) | 1.02 | (0.97, 1.08) | |
| Fourth quartile | 1.01 | (0.97, 1.05) | 1.09** | (1.03, 1.16) | 0.99 | (0.95, 1.04) | 1.05 | (0.9, 1.10) | |
| Nulliparous  (ref: multiparous) | 1.01 | (0.98, 1.03) | 0.95* | (1.03, 1.13) | 0.99 | (0.96, 1.03) | 0.79** | (0.76, 0.82) | |
| Facility delivery  (ref: at home/ other) | 2.09** | (2.03, 2.15) | 23.60** | (21.06, 26.48) | 0.23** | (0.22, 0.24) | 1.87** | (1.80, 1.94) | |

*p<0.05

**p<0.01

**Supplementary Figure 1.**

**Supplementary Table 3. Adjusted mortality by essential element for all infants, survival > 3 hours**

| Indicator | aHR | 95% CI | aHR | 95% CI | aHR | 95% CI | aHR | | 95% CI |
| --- | --- | --- | --- | --- | --- | --- | --- | --- | --- |
|  | **Immediate and thorough drying** | | **Skin to skin contact** | | **Delayed cord clamping^b^** | | **Early initiation of breastfeeding^b^** | | |
| Essential element | 1.09 | (0.93, 1.27) | 0.64** | (0.51, 0.81) | 0.87 | (0.66, 1.14) | 0.72** | (0.60, 0.87) | |
| Sex of infant  (ref: male) | 1.00 | (0.86, 1.17) | 0.97 | (0.84, 1.13) | 1.10 | (0.95, 1.29) | 1.04 | (0.90, 1.20) | |
| Preterm birth  (ref: >37 weeks) | 2.92** | (1.50, 3.40) | 2.88** | (2.48, 3.35) | 1.71** | (1.46, 2.01) | 1.70** | (1.46, 1.97) | |
| Mother’s literacy  (ref: illiterate) | 0.75** | (0.62, 0.91) | 0.78* | (0.65, 0.94) | 0.86 | (0.71, 1.05) | 0.90 | (0.75, 1.08) | |
| SES score  (ref: first quartile) |  |  |  |  |  |  |  |  | |
| Second quartile | 0.95 | (0.78, 1.16) | 0.98 | (0.81, 1.20) | 1.04 | (0.85, 1.28) | 1.03 | (0.85, 1.24) | |
| Third quartile | 0.80* | (0.64, 0.99) | 0.83 | (0.67, 1.03) | 0.93 | (0.75, 1.17) | 0.95 | (0.77, 1.18) | |
| Fourth quartile | 0.90 | (0.72, 1.12) | 0.89 | (0.71, 1.10) | 0.97 | (0.78, 1.22) | 0.98 | (0.79, 1.21) | |
| Nulliparous  (ref: multiparous) | 1.60** | (1.36, 1.88) | 1.57** | (1.34, 1.84) | 1.46** | (1.24, 1.72) | 1.36** | (1.17, 1.60) | |
| Facility delivery  (ref: at home/ other) | 1.15 | (0.98, 1.36) | 1.44** | (1.22, 1.71) | 0.77* | (0.59, 0.99) | 0.93 | (0.80, 1.08) | |

*p<0.05

**p<0.01

^b^stratified by follow up less than or greater than three days

**Supplementary Table 4. Adjusted mortality by essential element for all infants, survival > 48 hours^b^**

| Indicator | aHR | 95% CI | aHR | 95% CI | aHR | 95% CI | aHR | | 95% CI |
| --- | --- | --- | --- | --- | --- | --- | --- | --- | --- |
|  | **Immediate and thorough drying** | | **Skin to skin contact** | | **Delayed cord clamping** | | **Early initiation of breastfeeding** | | |
| Essential element | 1.12 | (0.90, 1.39) | 0.66** | (0.49, 0.90) | 1.07 | (0.75, 1.53) | 0.80 | (0.63, 1.00) | |
| Sex of infant  (ref: male) | 1.07 | (0.87, 1.31) | 1.02 | (0.84, 1.25) | 1.05 | (0.86, 1.29) | 0.99 | (0.82, 1.22) | |
| Preterm birth  (ref: >37 weeks) | 2.09** | (1.68, 2.59) | 2.06** | (1.67, 2.55) | 2.08** | (1.68, 2.58) | 2.10** | (1.71, 2.58) | |
| Mother’s literacy  (ref: illiterate) | 0.77* | (0.59, 0.99) | 0.80 | (0.62, 1.03) | 0.79 | (0.61, 1.02) | 00.82 | (0.64, 1.05) | |
| SES score |  |  |  |  |  |  |  |  | |
| First quartile | Ref | Ref | Ref | Ref | Ref | Ref | Ref | Ref | |
| Second quartile | 1.09 | (0.84, 1.42) | 1.09 | (0.84, 1.42) | 1.10 | (0.85, 1.44) | 1.10 | (0.85, 1.42) | |
| Third quartile | 0.83 | (0.61, 1.12) | 0.89 | (0.66, 1.20) | 0.84 | (0.62, 1.14) | 0.88 | (0.66, 1.18) | |
| Fourth quartile | 0.95 | (0.70, 1.27) | 0.96 | (0.71, 1.29) | 0.97 | (0.72, 1.30) | 0.91 | (0.68, 1.22) | |
| Nulliparous  (ref: multiparous) | 1.74** | (1.39, 2.17) | 1.71* | (1.37, 2.13) | 1.74** | (1.40, 2.17) | 1.62** | (1.31, 2.02) | |
| Facility delivery  (ref: at home/ other) | 0.88 | (0.71, 1.11) | 1.12 | (0.89, 1.42) | 0.99 | (0.70, 1.39) | 1.01 | (0.83, 1.28) | |

*p<0.05

**p<0.01

^b^stratified by follow up less than or greater than three days

**Supplemental Table 5. Correlation matrix**

|  | **Immediate and thorough drying** | **Delayed cord clamping** | **Skin to skin contact** | **Early initiation of breastfeeding** |
| --- | --- | --- | --- | --- |
| **Immediate and thorough drying** | 1.000 |  |  |  |
| **Delayed cord clamping** | -0.3133* | 1.000 |  |  |
| **Skin to skin contact** | 0.1037* | -0.4911* | 1.000 |  |
| **Early initiation of breastfeeding** | 0.1040* | -0.1665* | 0.1493 | 1.000 |

*****p-value <0.01

**Supplemental Table 6. All elements adjusted for covariates^b^ and other elements, by survival time**

|  | Survival >3 hours | | Survival > 48 hours | |
| --- | --- | --- | --- | --- |
|  | **aHR** | **95% CI** | **aHR** | **95% CI** |
| Immediate and thorough drying | 1.09 | 0.93, 1.30 | 1.14 | 0.91, 1.42 |
| Skin to skin contact | 0.72** | 0.57, 0.92 | 0.70* | 0.51, 0.96 |
| Delayed cord clamping | 0.89 | 0.67, 1.18 | 1.04 | 0.72, 1.52 |
| Early initiation of breastfeeding | 0.80* | 0.66, 0.98 | 0.85 | 0.66, 1.09 |

^b^covariates (preterm birth, sex of infant, mother’s literacy, SES, parity and place of delivery)

**Supplemental Table 7. Adjusted mortality for all infants > 3 hours postpartum, by preterm birth**

|  | Gestational Age >37 weeks | | | | Gestational Age < 37 weeks | | | |
| --- | --- | --- | --- | --- | --- | --- | --- | --- |
|  | **Live births** | **Deaths** | **aHR** | **95% CI** | **Live births** | **Deaths** | **aHR** | **95% CI** |
| Immediate and thorough drying | 21690 | 403 | 1.21 | 0.98, 1.49 | 5075 | 277 | 0.92 | 0.71, 1.19 |
| Skin to skin contact | 22035 | 418 | 0.59** | 0.44, 0.79 | 5165 | 286 | 0.76 | 0.52, 1.09 |
| Delayed cord clamping | 21620 | 397 | 0.80 | 0.56, 1.14 | 5061 | 271 | 0.94 | 0.60, 1.45 |
| Early initiation of breastfeeding | 22665 | 442 | 0.65** | 0.51, 0.83 | 5314 | 306 | 0.84 | 0.63, 1.14 |

^b^covariates (PTB, sex of infant, mother’s literacy, SES, parity and place of delivery)

*p<0.05

**p<0.01

**Supplemental Figures 1a-1d**

**2a**

**2b**

**2c**

**2d**

**Supplemental table 8. Characteristics of mother-infant dyads with “missing” element information**

|  | Immediate and thorough drying | | Skin to skin contact | | Delayed cord clamping | | Early initiation of breastfeeding | |
| --- | --- | --- | --- | --- | --- | --- | --- | --- |
|  | Not missing | Missing | Not missing | Missing | Not missing | Missing | Not missing | Missing |
| Child gender |  |  |  |  |  |  |  |  |
| Male | 51.51% | 54.91% | 51.55% | 55.45% | 51.57% | 53.55% | 51.64% | 65.79% |
| Female | 48.39% | 45.09% | 48.45% | 44.55% | 48.43% | 46.45% | 48.36% | 34.21% |
| PTB |  |  |  |  |  |  |  |  |
| >37 weeks | 81.04% | 80.10% | 81.02% | 80.51% | 81.04% | 80.30% | 81.01% | 72.97% |
| < 37 weeks | 18.96% | 19.90% | 18.98% | 19.49% | 18.96% | 19.70% | 18.99% | 27.03% |
| Mother’s literacy |  |  |  |  |  |  |  |  |
| Illiterate | 69.48% | 50.84% | 69.26% | 48.23% | 69.62% | 49.29% | 68.64% | 78.95% |
| Literate | 30.52% | 49.16% | 30.74% | 51.77% | 30.38% | 50.71% | 31.36% | 21.05% |
| SES quartile |  |  |  |  |  |  |  |  |
| 1 | 34.73% | 22.11% | 34.57% | 20.93% | 34.87% | 20.16% | 34.17% | 31.58% |
| 2 | 21.67% | 18.75% | 21.65% | 17.87% | 21.64% | 19.49% | 21.52% | 34.21% |
| 3 | 20.46% | 21.95% | 20.48% | 22.03% | 20.41% | 23.00% | 20/53% | 18.42% |
| 4 | 23.14% | 37.19% | 23.30% | 39.17% | 23.09% | 37.34% | 23.78% | 15.79% |
| Parity |  |  |  |  |  |  |  |  |
| Nulliparous | 72.67% | 56.58% | 72.46% | 54.96% | 72.81% | 54.89% | 71.95% | 76.32% |
| Multiparous | 27.33% | 43.42% | 27.54% | 45.04% | 27.19% | 45.11% | 28.05% | 23.68% |
| Facility delivery |  |  |  |  |  |  |  |  |
| No | 60.15% | 6.70% | 59.38% | 3.92% | 60.51% | 3.06% | 57.75% | 73.68% |
| Yes | 39.85% | 93.30% | 40.62% | 96.08% | 39.49% | 96.94% | 42.25% | 26.32% |
